# Relationship between sex biases in gene expression and sex biases in autism and Alzheimer’s disease

**DOI:** 10.1186/s13293-024-00622-2

**Published:** 2024-06-07

**Authors:** Stuart B. Fass, Bernard Mulvey, Rebecca Chase, Wei Yang, Din Selmanovic, Sneha M. Chaturvedi, Eric Tycksen, Lauren A. Weiss, Joseph D. Dougherty

**Affiliations:** 1 Department of Genetics, Washington University School of Medicine, 660 S. Euclid Ave, Saint Louis, MO 63110 USA; 2 Department of Psychiatry, Washington University School of Medicine, 660 S. Euclid Ave, Saint Louis, MO 63110 USA; 3 Lieber Institute for Brain Development, 855 North Wolfe St. Ste 300, Baltimore, MD 21205 USA; 4 McDonnell Genome Institute, Washington University School of Medicine, St. Louis, MO 63110 USA; 5 Intellectual and Developmental Disabilities Research Center, Washington University School of Medicine, 660 S. Euclid Ave, Saint Louis, MO 63110 USA; 6 Institute for Human Genetics, University of California, San Francisco, 513 Parnassus Ave, HSE901, San Francisco, CA 94143 USA; 7 Department of Psychiatry and Behavioral Sciences, University of California, San Francisco, 513 Parnassus Ave, HSE901, San Francisco, CA 94143 USA; 8 Weill Institute for Neurosciences, University of California, San Francisco, 513 Parnassus Ave, HSE901, San Francisco, CA 94143 USA; 9 Department of Genetics, 4566 Scott Ave., Campus Box 8232, St. Louis, MO 63110-1093 USA

**Keywords:** Sex-bias, Sex, Expression, rna-seq, Alzheimer’s, Autism, Neuronal, Immune, Brain, Human

## Abstract

**Background:**

Sex differences in the brain may play an important role in sex-differential prevalence of neuropsychiatric conditions.

**Methods:**

In order to understand the transcriptional basis of sex differences, we analyzed multiple, large-scale, human postmortem brain RNA-Seq datasets using both within-region and pan-regional frameworks.

**Results:**

We find evidence of sex-biased transcription in many autosomal genes, some of which provide evidence for pathways and cell population differences between chromosomally male and female individuals. These analyses also highlight regional differences in the extent of sex-differential gene expression. We observe an increase in specific neuronal transcripts in male brains and an increase in immune and glial function-related transcripts in female brains. Integration with single-nucleus data suggests this corresponds to sex differences in cellular states rather than cell abundance. Integration with case–control gene expression studies suggests a female molecular predisposition towards Alzheimer’s disease, a female-biased disease. Autism, a male-biased diagnosis, does not exhibit a male predisposition pattern in our analysis.

**Conclusion:**

Overall, these analyses highlight mechanisms by which sex differences may interact with sex-biased conditions in the brain. Furthermore, we provide region-specific analyses of sex differences in brain gene expression to enable additional studies at the interface of gene expression and diagnostic differences.

**Graphical Abstract:**

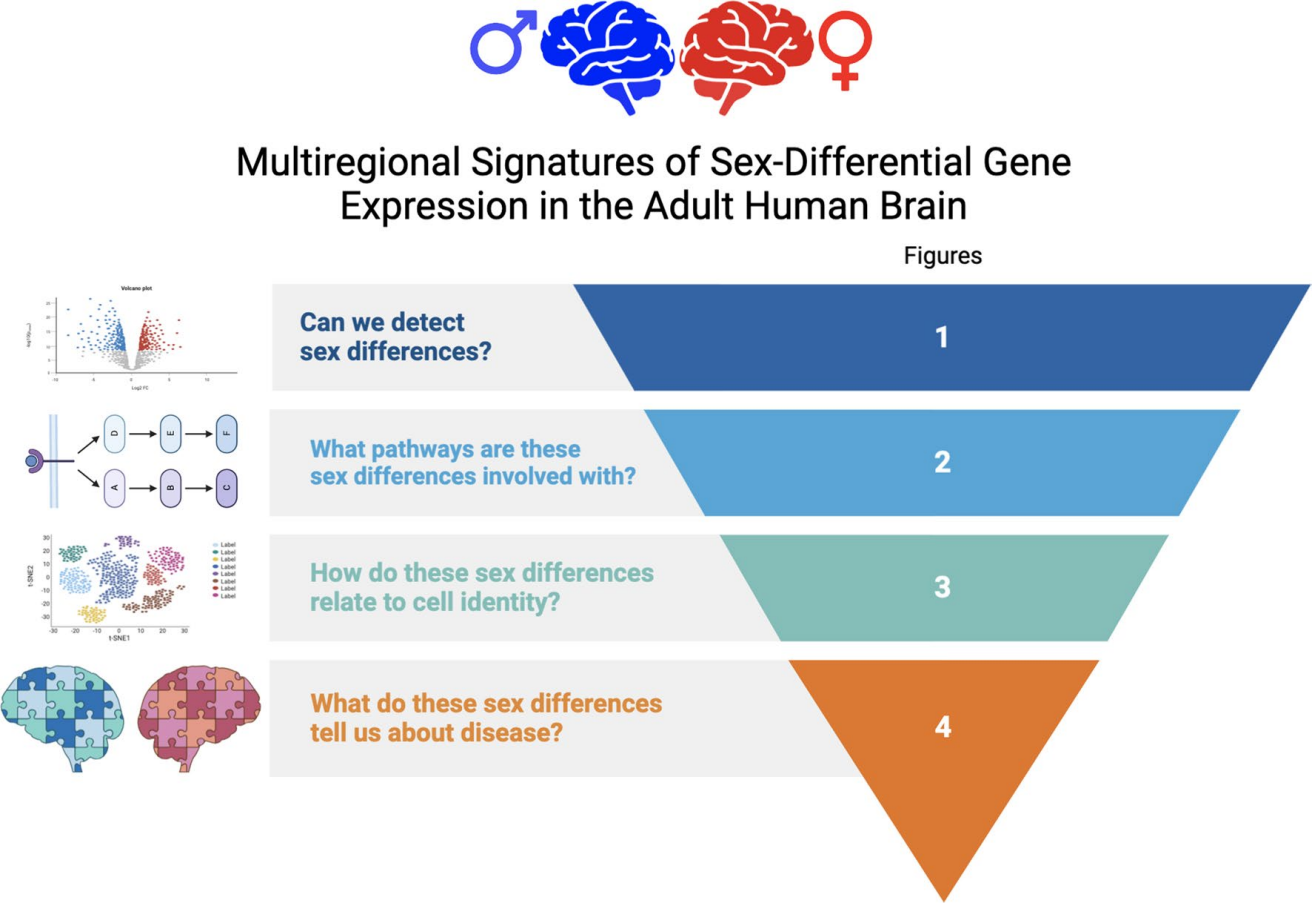

**Supplementary Information:**

The online version contains supplementary material available at 10.1186/s13293-024-00622-2.

## Highlights

- Numerous but small autosomal sex differences in expression exist in all brain regions tested.
- Autosomal genes with enriched expression in males are enriched in neuronal pathways.
- Autosomal genes with enriched expression in females are enriched with immune system functions.
- Integration with single-nucleus datasets suggest these differences are more likely related to cell state differences than cell number differences.
- The female cortex shows an enrichment of genes expressed in Alzheimer’s disease brains.

**Supplementary Information:**

The online version contains supplementary material available at 10.1186/s13293-024-00622-2.

## Abstract (plain language summary)

We sought to understand why females have higher rates of Alzheimer’s disease, and males have higher rates of autism. One idea was that the female brain at baseline may be more similar to an Alzheimer’s brain, so it is easier for them to shift into that state (likewise, males may be more similar to autism). To test this, we examined gene expression differences between brains of biological males and biological females. While all people have the same ~ 25,000 genes, each gene can be on or off (‘expressed’) to different extents. Overall, we found that there were differences in gene expression between males and females in all brain regions tested but more differences in some brain regions than others. By looking at the role of these genes we estimate that female immune system processes might be more active in the brain. We also found female brain gene expression looked slightly more like people with Alzheimer’s compared to people without Alzheimer’s, which may explain why females get Alzheimer’s disease more easily. However, the male brain gene expression did not look more like autism, suggesting that the reason males have higher rates of autism is complex and needs further investigation.

**Supplementary Information:**

The online version contains supplementary material available at 10.1186/s13293-024-00622-2.

## Background

Most human neuropsychiatric conditions show differences in diagnostic rates between males and females. For example, males make up a higher percentage of diagnosed neurodevelopmental conditions that begin in early life, such as autism and Attention Deficit-Hyperactivity Disorder (ADHD). Conversely, females are more likely to suffer later-onset disorders such as Major Depressive Disorder and Alzheimer’s disease [1, 2]. Even though a major component of risk for these neuropsychiatric conditions is heritable, genetic risk is complex, with hundreds to thousands of genes and variants implicated [3–13]. Depending on sex, manifestations of many disorders differ molecularly [14–16] and in their clinical presentation [17–19]. Overall, the multifactorial heritability patterns and heterogenous phenotypes of neuropsychiatric conditions have been a substantial barrier toward understanding the biological processes governing sex variation in risk and presentation.

Each of these sex-biased conditions is strongly influenced by large numbers of common, non-coding variants in the genome [3, 5–9, 11]. Common variants are thought to influence risk of psychiatric conditions by subtly affecting the expression of nearby genes in the brain. These many small changes in gene expression can, in aggregate, greatly alter risk of presenting with a given condition [20–22], thus giving gene expression an important role in pathogenesis and progression.

Sex differences in transcription are modulated by several classes of DNA-interacting proteins, some of which are encoded on allosomes (X and Y chromosomes). Some DNA-interacting proteins are also modulated by sex-differential hormonal signals, including androgens, estrogens, and progestins. Beyond the direct binding sites of allosome-encoded proteins or sex hormone receptors, these modulatory factors appear to have prominent roles in sex-differential transcriptional regulation via indirect and co-regulatory activity, including at-risk variants for sex-biased psychiatric conditions [23–27]. Ultimately, understanding the full extent of how and where sex governs transcription will improve the understanding of how gene expression affects the odds of developing a particular neuropsychiatric condition, the condition’s presentation, and how sex might relate to genetic expression differences.

Autism is particularly illustrative of the complex interaction between sex and genetic risk. Researchers have proposed that subtle baseline sex differences in gene expression can shift the brain towards a transcriptomic signature that might promote the condition in a particular sex [16, 28]—i.e., perhaps the male brain at baseline has a ‘molecular predisposition’ towards autism, and thus it takes fewer heritable genetic or environmental factors to meet criteria for a diagnosis, as posited by the “extreme male brain” theory [29]. If indeed case–control differences in transcription highlight such a state, a prediction of this model is that transcription in the male brain at baseline will be more similar to what is seen in postmortem autism brains. Indeed, past work found that upregulated genes in the neurotypical postmortem male (vs. female) cortex are more highly expressed in the postmortem cortex of autism patients from both sexes when compared to controls [30].

In addition to the effects of common variants on risk, rare loss of function mutations—implying a 50% reduction in expression—also cause disease, particularly for syndromic forms of autism and Intellectual Disability where hundreds of new causal genes have recently been identified [31, 32]. Thus, it would be interesting to examine whether any sex biases in gene expression overlap with rare variant disorder genes. Previous work found that genes implicated in rare variant forms of autism at that time did not show any sex bias in expression [30].

Therefore, to replicate and extend these prior studies, we further characterized the transcriptomes of adult brains using larger datasets and additional brain regions, and tested whether sex-differential expression (sex-DE) of risk genes themselves may underlie sex differences in incidence of two prominent sex-biased conditions—one male-biased (autism) and one female-biased (Alzheimer's disease), both of which were selected because they have robust genome-wide association studies (GWAS) and case–control gene expression data. We examined two of the largest collections of postmortem brain RNA sequencing (RNA-seq) data available: GTEx version 8 [33] and the CommonMind Consortium (CMC) [34]. A key advantage of the GTEx dataset is that it surveys multiple brain regions across hundreds of male and female individuals, enabling an analysis for sex both within and across brain regions. The CMC dataset consists of only frontocortical samples, which we used to benchmark our analysis of GTEx cortex and produce a high-confidence meta-analyzed set of sex-differentially expressed cortical genes. We thus present a resource grounded in a large bulk RNA-Seq brain dataset, detailing sex-differential expression in the human adult brain at both broad and fine scale. Using signal-to-noise ratio (SNR) analyses, we identify regions with the most robust transcriptome-wide DE signatures within the GTEx data. We then identify differentially expressed genes (DEGs) in a novel ‘omnibus’ brain-wide framework, as well as DEGs for each region individually. With our omnibus analysis we identify a substantial proportion of the transcriptome as being sex-DE, albeit at very small magnitudes. From omnibus and regional sex DEGs, we then identify pathways and cell types over- or under-represented in each sex. We integrate these results with insights from recent human single-nucleus RNA-Seq (snRNA-seq) data, which provide more refined cell type and subtype gene signatures. Finally, we examine whether baseline sex DE overlaps with rare and common variant disease-associated gene sets and DEGs from postmortem human brain studies of neuropsychiatric cohorts.

## Methods

### Data pre-processing, filtering, and normalization

For this work, we utilized bulk RNA-sequencing data previously conducted by GTEx and the Commonmind Consortium Specifically, we used GTEx project v8 release gene count data (annotated with Gencode v26). Sample and donor attribute files were downloaded from dbGaP( phs000424.v9.p2). CMC data was downloaded from the CMC Knowledge Portal (10.7303/syn2759792) and annotated with GRCh37. Except where noted, the GTEx and CMC data were handled identically.

All donors coded either as having an unknown status or positive diagnosis for brain-related diseases were removed to avoid confounding variables in the data. Removed brain-related diseases included amyotrophic lateral sclerosis, Alzheimer’s disease, dementia, encephalitis at death, Creutzfeld–Jakob disease, multiple sclerosis, Parkinson’s disease, Reye’s syndrome, and schizophrenia. We additionally removed donors positive or of unknown status for major systemic diseases with potential to impact the brain secondarily in line with prior analyses [35]. This included sepsis/positive blood cultures, lupus, cardiovascular disease, human immunodeficiency virus, active cancer diagnosis, high unexplained fever, abnormal white blood cells, influenza, and opportunistic infections. Overall this resulted in the sample numbers and sex distribution described in Table 1. For the *n* = 1688 samples retained from these donors, the average RNA integrity number (RIN) was 6.95. The R package *edgeR* was used to filter out low-representation genes, retaining only those with greater abundance than 10 counts per million (CPM) in at least 19 samples: a cutoff determined by taking 70% of the smallest group size of 27 (female amygdala). Data was then weighted and scaled by library size with the trimmed mean of m-values (TMM) method. For the CMC dataset, all samples with a Schizophrenia or Klinefelter diagnosis were removed from the analysis. Overall this resulted in the sample numbers and sex distribution described in Table 1.

**Table 1:** Donor demographic and sample quality information from GTEx and CMC datasets

**GTEx (N = 1688)**	
* Sex*	
Male	1230 (72.9%)
Female	458 (27.1%)
* Brain region*	
Amygdala	91 (5.4%)
Anterior Cingulate Cortex	104 (6.2%)
Caudate	154 (9.1%)
Cerebellum	298 (17.7%)
Cortex	297 (17.6%)
Hippocampus	134 (7.9%)
Hypothalamus	126 (7.5%)
Nucleus Accumbens	160 (9.5%)
Putamen	135 (8.0%)
Spinal cord	104 (6.2%)
Substantia Nigra	85 (5.0%)
* Age (years)*	
Mean (standard deviation)	57.0 (10.8)
Median [min, max]	59.0 [20.0, 70.0]
* Age bracket*	
20’s	70 (4.1%)
30’s	52 (3.1%)
40’s	164 (9.7%)
50’s	576 (34.1%)
60’s	723 (42.8%)
70’s	103 (6.1%)
*RIN*	
Mean (standard deviation)	6.95 (0.855)
Median [min, max]	6.80 [5.00, 10.0]

**CMC (N = 437)**	
* Sex*	
Male	278 (63.6%)
Female	159 (36.4%)
Brain region	
Cortex	437 (100%)
* Age (years)*	
Mean (standard deviation)	57.8 (21.5)
Median [min, max]	57.0 [18.0, 108]
* Age bracket*	
10’s	12 (2.7%)
20’s	40 (9.2%)
30’s	34 (7.8%)
40’s	76 (17.4%)
50’s	76 (17.4%)
60’s	64 (14.6%)
70’s	50 (11.4%)
80’s	48 (11.0%)
90’s	33 (7.6%)
100’s	4 (0.9%)
* RIN*	
Mean (standard deviation)	7.78 (0.891)
Median [min, max]	7.90 [4.50, 9.30]

### Surrogate variable (SV) analysis

Given the broad number of epidemiologic variables in the GTEx cohort, surrogate variables (SVs) were included to account for unknown latent sources of variation in the data. Forty nine SVs were identified using the R package *sva* [36]. The full model and null model used for this analysis are as follows:

$$full= \sim 0 + SEX\_REGION$$

$$null= \sim 1$$

For the CMC dataset, we identified SVs representing latent sources of variation, all 9 of which were included in the DE model. All SVs used in the analysis along with the full design are included in Supplemental Table 8.

### QC

To perform quality checks on the data, principal component analysis (PCA) plots were generated and the effect of SVs were visualized using the R package *limma* [37, 38]. Then, SVs were tested for correlation with any sex-region group in the data. Distributions of PCs generated from the counts matrix were plotted before and after SV correction to ensure SVs were not erroneously grouping samples or creating outliers. Mean–variance trends were plotted and inspected visually to ensure genes were following typical trends for a sizable multigroup RNA-Seq experiment. Specifically, we wanted to ensure there were no outlier genes relative to the mean–variance trend line, and that genewise dispersions were in the 0.5 to 1.5 range (Supplemental Fig. 1).

### Signal to noise ratio

Due to the high degree of variability inherent in obtaining postmortem brain tissue, it was critical to determine whether the influence of our biological signal of interest (sex) was detectable over technical noise. To evaluate this, we compared the total differences between males and females to the total variance in the unadjusted data as outlined by Lopes-Ramos [39]. This method allowed us to calculate a signal-to-noise ratio (SNR). The exact equation for SNR calculations are as follows:

$$\begin{aligned} tSNR\left( {X,Y} \right) & = \frac{{\left\| {\overline{X} - \overline{Y}} \right\|_{2} }}{{\sqrt {\frac{{\sigma_{X}^{2} }}{F} + \frac{{\sigma_{Y}^{2} }}{M}} }} \\ \sigma_{X}^{2} & = \frac{{\sum\nolimits_{i = 1}^{F} {\left\| {X_{i} - \overline{X}} \right\|_{2}^{2} } }}{F - 1} \\ \sigma_{Y}^{2} & = \frac{{\sum\nolimits_{i = 1}^{M} {\left\| {Y_{i} - \overline{Y}} \right\|_{2}^{2} } }}{M - 1} \\ \end{aligned}$$

Let F denote the number of females and M denote the number of males, and let X and Y be the matrices of gene expression values in females and males respectively, and let $\underline{X}$ and $\underline{Y}$ be the genewise expression average across all female and male samples. In order to determine SNR values without bias for unbalanced representation of sexes in the data, we created both a “true” and a “null” distribution. To generate the “true” distribution we calculated the SNR 10,000 times based on randomly drawn samples with correct sex labels to generate a “true” distribution, Then, to generate the “null” distribution we calculated the above value 10,000 more times from randomly drawn samples that were split into two arbitrary groups and randomly assigned a sex to create a null distribution. For each iteration per region, where n = 90% of the smaller (in all cases, female) group size, *n* male samples and *n* female samples were drawn (or for null simulations, 2n of the total samples). For a hypothetical brain region with 200 male samples and 100 female samples, 90 male and 90 female samples would be randomly selected per ‘true’ iteration and 180 samples selected and randomly assigned a sex per null iteration. We compared the distributions with Wilcox tests, and also calculated empirical P values as follows:

$$P = (SNR_{null} > SNR_{true} ) / n_{iterations}$$

### Differential expression analysis

Linear model designs were created using each sex-region (group) and the remaining SVs as fixed effects and donor as a random effect. Note that random effects (otherwise known as blocking factors) were estimated using a parallel implementation of the *limma* function *duplicateCorrelation()* (see *Code and Modules below*).

$$MOD = \sim 0 + SEX\_REGION + SV1 \cdots + SV49 + (1|Donor)$$

After the model was created, the count matrix was transformed to moderated log_2_(CPM) with a parallelized implementation of *limma’s VoomWithQualityWeights()* function. These functions fit the counts matrix to a linear model estimating for random factors, and adjust the relative weight of each sample to the mean variance of the sample. In short, this means that samples with high variance relative to the mean variance of all the samples will have less weight when detecting differentially expressed (DE) genes. This is a good replacement for trying to model the quality of the samples with RIN scores, which is generally a poor estimator [40]. The linear “mixed” model is then fit to the adjusted data. Our dataset-wide modeling strategy allows for regional and omnibus contrasts using the same model, yielding regionally comparable results and precluding the need for multiple-testing correction for multiple contrasts. The following contrasts were used:

$$Region = Female\, Region - Male \,Region$$

$$Omnibus = \left( {Female\, Regions - Male \,Regions} \right) / n\, Regions$$

Therefore, all positive logFC values reported indicate greater expression in females relative to males, and all negative logFC values indicate greater expression in males versus females. The linear model-adjusted data are then contrasted to calculate differential expression. Finally the data are smoothed using an empirical Bayes method, which squeezes the genewise-wise residual variances of the data towards a common value and provides a better estimate of the t-statistic than an unmoderated version. The DE tables for each region and the omnibus contrast are provided in Supplemental Table 1.

A slight adjustment was made for the CMC dataset since there were fewer overall samples and all samples were from a single brain region. First, limma’s pre-packaged functions were sufficient for this model. Second, without the region variable our only biological factor of interest was sex resulting in the following model:

$$MOD = \sim 0 + SEX + SV1 \cdots SV9$$

The DE table for the CMC data is available in Supplemental Table 2.

### Gene set enrichment analysis

In order to test for categories of biological function enriched in sex-differentially expressed genes outside of sex chromosomes, we performed Gene Set Enrichment Analysis (GSEA). All autosomal protein-coding genes with detectable brain signal, and their log2FCs, were used as input for analysis with the GSEA tool (version 4.2.3) [41, 42]. 1000 permutations were used and gene sets were restricted to those between 15–500 genes in size. Full list of results are available in Supplemental Table 3.

### *Meta*-analysis

To get a better picture of the sex-differential biology we combined the results of the two independent analyses through a meta analysis. To do this we first took the intersection of the two datasets’ genes using their Ensembl ID’s, then removed all genes that didn't agree on the direction of logFC effect. Once we had our list of genes we combined their P-values using the *AWFisher* R package [43], which utilizes adaptively weighted log-p values from individual studies to generate a unified statistic and the associated p-value for the significance of the combined result. Once the P values were calculated they were subsequently adjusted using the Benjamini–Hochberg method, otherwise known as FDR correction. Results are available in Supplemental Table 4.

### ChIP-X enrichment analysis (ChEA3)

In order to better understand which transcription factors (TFs) may be responsible for regional sex DEGs we conducted an analysis of TF using the ChEA3 tool [44]. We first took all TFs that ranked 50 or lower using the mean rank metric. To avoid circular logic we excluded the GTEx coexpression heuristic from the ChEA3 tool results and recalculated the mean ranks from the other four heuristics. For increased rigor, TFs also had to also be significantly DE in the tissue of DEGs entered into the ChEA3 tool, meaning that the TFs were subset only to activators. For a complete list of TFs that met these criteria see Supplemental Table 5.

### Single-nucleus enrichment analysis of genes upregulated in cortexfor each sex

The Allen Brain Atlas provides a 47,000 nucleus, single-nucleus RNA-seq dataset from 6 areas of cortex representing all major cortical cell types [45]. We utilized this dataset to identify cell types/states for which there was more sex-biased differential gene expression. For each nucleus we calculated a single-cell disease relevance score (scDRS) with the scDRS tool from Zhang et al. with the following parameters: mean–variance based control gene selection, 1500 permuted controls per cell, and low-count/low-gene-total pre-filtering [46]. Sets of sex-biased DEGs for input were generated from the top 1000 autosomal genes upregulated in male OR female cortex, for a total of 2 gene score sets. Genes are weighted by a Z-score, in this case, the Z-normalized DE significance. Subsequently, the scDRS tool’s downstream functions were utilized to identify genes most correlated with the weighted signatures and quantify enrichment significance and heterogeneity defined as variability in enrichment scores within pre-labeled groups, in cell types and cortical layers.

### Comparison to genes near associated common variants from genome-wide association studies for autism, ADHD, and AD

Candidate genes based on proximity to genome-wide association study (GWAS) peak and transcriptome-wide association (TWAS) analysis were collected for GWAS loci associated with ADHD and autism when considered jointly [12]. TWAS genes with P < 0.05 were retained for list overlap. For AD, previously identified genes/GWAS loci were collected from a recent Alzheimer’s GWAS [11]. From this study, we combined gene lists identifying known Alzheimer's risk genes and newly discovered risk genes. DEGs from each sex for each GTEx region were overlapped with the autism/ADHD and Alzheimer’s gene sets described above. A Fisher’s test was used to calculate the enrichment of sex DEGs among putative GWAS target genes.

To examine whether sex-differentially expressed Alzheimer's genes from GTEX cortex were targets of particular mature microRNAs (miRNAs), miRDB [46] was used to retrieve all miRNAs predicted to regulate mRNA level for the overlapping FDR-significant genes upregulated in each sex. These predictions were then subsetted to those miRNAs retained for expression in our analysis (13 miRNAs total) and used to generate the regulatory network (Fig. 4D) via Cytoscape [47].

### Overlap of sex DE genes with genes associated with autism by rare variant studies, and with genes differentially expressed in cases and controls

In order to examine how our results may relate to sex-biased disease, we compared our results with those from prior autism and Alzheimer’s-oriented studies. For autism rare variant genes we used SFARI Genes specifically ‘genescore 1’ genes, which are genes with the highest level of evidence supporting their role in autism [31]. For autism case–control DEG sets we used two prior studies, Gandal supplementary Table 3 [48] and Werling supplemental Table 2, specifically Voineagu autism up and down regulated DEGs(sheets 5 and 6) [30, 49]. For Alzheimer’s case–control gene sets we used the RNA-Seq Harmonization Study [50], specifically the cortical region contrasts. We then subset the lists from prior studies to only contain genes that were included in our analysis, and split the lists based on direction of effect (case upregulated, control upregulated). We then tested these multiple gene sets for enrichment in each of our sex by region DEG sets by Fisher test.

### CPM match enrichment permutation test

To test whether the overlap between male-biased DEGs and rare causal variants in autism were not simply explained the generally increased levels of neuronal transcripts in the male samples, we tested whether random genes with similar expression in neurons would show similar enrichment to SFARI genescore 1 genes, using an approach derived from [51], but updated to use single cell neuronal data to generate the random gene lists. Specifically, we utilized the Allen Brain Atlas cortex postmortem single-nucleus RNA data [45] and subsetted it to include only neuronal cell types. In short, we generated a log(CPM) value for each neuronal gene and created individual probability matrices. This was then compared to probability matrices for only genes categorized as SFARI genescore 1. Permutations were performed 1000 times and each gene set was tested for enrichment in the male cortex DEG set. Finally, we plotted the Odds Ratio (OR) of all 1000 permuted gene sets along with the real OR from the genuine SFARI genescore 1 genes, and calculated an empirical P value for the true set based on its placement in the random distribution.

### Code and modules

Functions from multiple sources,as well as custom code were used to run this analysis. Code has been deposited into Bitbucket at https://bitbucket.org/jdlabteam/gtex_peper_final_code/src/main/.

## Results

### Processing of RNA sequencing data identifies a sex signal in most brain regions

**Fig. 1:**
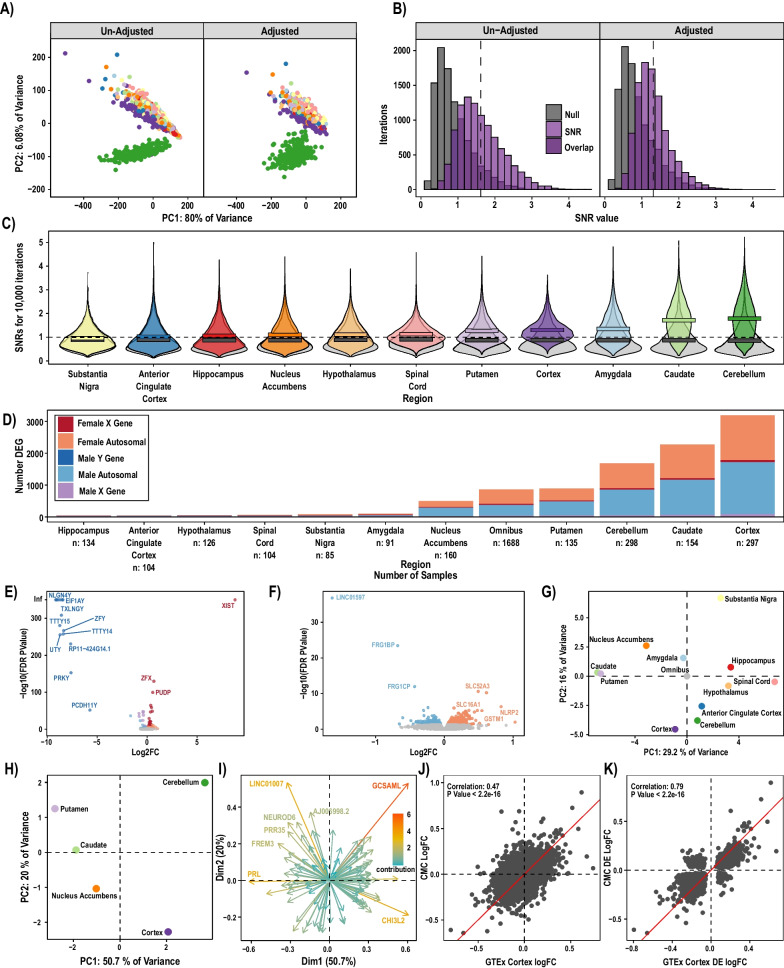
Brain regions show distinct patterns of sex-differential expression. **A** PCA plot of all samples and genes before and after removing the effects of SVs. Accounting for SV’s reduces the variance in the data, and cerebellar samples (green) cluster separately from other regions (*colors as in C*). **B** SNR from 10,000 iterations, pre- and post-SV adjustment in cortex. SV adjustment reduces the tail of the distribution, makes the distribution more normal, and reduces mean SNR. **C** SV-adjusted SNR values (10,000 iterations). Each distribution is significantly different from its corresponding null distribution by Wilcox test. All regions other than substantia nigra have a mean SNR value greater than one, providing evidence that there is an expression difference between males and females in multiple brain regions. Cortex has the third highest mean SNR value and has the shortest tail, suggesting that its SNR is highly repeatable. **D** Summary of DE gene count per region, including omnibus. There is an abundance of DE autosomal genes in the nucleus accumbens, cortex, cerebellum, putamen, and caudate. Sample number does not fully explain the number of DE genes in a given region. **E** Volcano plot highlighting that allosomal genes follow expected trends. **F** Volcano plot highlighting the autosomal genes, including noteworthy long non-coding RNA *LINC01597.* **G** PCA of all LogFCs from all regions and omnibus, shows omnibus truly represents the average sex-differential expression across all brain regions. **H** PCA of top 500 most variable LogFCs that were FDR significant in at least one of the “sex-differential” regions. Highlights the fact that cerebellum remains an outlier even when only considering sex differences. **I** PCA plots showing the key genes that separate the SDR in the same PCA space as panel H. Highlights a few notable genes that can be used to distinguish regions from a sex-differential lens. **J** Correlation of LogFCs between CMC, and GTEx analysis demonstrates results are robustly shared for the cortex. **K** Correlation of LogFCs for DE genes between CMC and GTEx shows a high replicability in cortex

We filtered and normalized bulk RNA-seq data from the GTEx project using a *limma* pipeline with models accounting for brain region, sex, surrogate variables (SVs), and donor effects. The full GTEXv8 release contains 2642 samples from 382 donors, spanning 13 brain regions (*n* per region ranging from 152 to 255 samples). The subset of GTEX donors included in our analyses (see “Methods”) contributed *n* = 1688 samples from seven regions per donor on average and were mostly aged 50 to 69 years (see Table 1 for additional demographic and sample information). After filtering, we analyzed **14082** genes, **497** of which were allosomal. PCA clustering separates tissues primarily by region (Fig. 1A), indicating this is the major mediator of gene expression differences across samples. We show that accounting for latent sources of variation in the data using SV adjustment does not produce outliers and largely acts to shrink the variance across samples (Fig. 1A).

### Sex-biased signal is stronger than technical noise in the majority of tissue samples

Differential expression analysis allows for identification of genes with sex-biased expression; however, a signal to noise ratio (SNR) analysis captures pan-transcriptomic divergence between groups without using arbitrary statistical thresholds and can aid data quality assessment by quantifying signal and variance in relative terms [39]. Moreover, this approach can be used to confirm noise-reducing effects of data pre-processing (low count removal, batch corrections, etc.) (Fig. 1B). We calculated SNR values for each region twice, once with unadjusted RNA counts and again with SV-adjusted counts. Random subsets of male and female samples were drawn 10^4^ times per region for both SV-adjusted counts and unadjusted counts (see “Methods”). We observed that adjusting for SVs makes the SNR distribution more similar to a normal distribution (Fig. 1B). When examining the SV-adjusted counts for all regions we saw that regions with larger total *n* (e.g. cerebellum) had the largest SNR, indicating either robust sex differences or exceptionally low noise due to statistical power (Fig. 1C). We also speculate that the large SNR might also be driven by ease of dissection, with the cerebellum and the cortex being relatively larger and easy to dissect compared to smaller brain structures such as the substantia nigra. The shape of the SNR distribution is also of importance as distributions approaching normal indicate that SNR values are replicable across sample subsets, while long-tailed distributions indicate that extreme findings can be driven by certain combinations of samples/donors (Fig. 1B). Unlike subsequent DE analysis, our SNR calculations did not account for random effects of donor. Substantia nigra showed a SNR of less than one, indicating that this region may not have sex-differentiated gene expression patterns, or is more difficult to dissect reproducibly and is thus noisier. For these reasons, we recommend caution in interpreting our sex DE findings from the substantia nigra. However, in remaining regions, a sex-biased signal greater than technical noise is evident, with the cortex standing out as a region with consistent and substantial sex differences.

### Sex differences in gene expression are widespread across brain regions

We next identified DE genes (FDR < 0.05) between males and females for each brain region, and across all regions in a general omnibus model (Fig. 1, Supplemental Table 2). Both autosomal and allosomal genes were included in illustrations and analyses except where noted. There are DEGs (FDR < 0.05) both on allosomes and autosomes (Fig. 1D). Many known X inactivation escape genes were found to be highly expressed in females, including *XIST*. As expected, DE genes shared across regions were allosomal: for example, 15% of allosomal genes (74, including all 14 chrY genes analyzed) were found to be DE in the omnibus model, consistent with base expectations of a sex DE analysis. In addition to the allosomal genes, we further identify a total of 5,182 unique autosomal genes DE in at least one of our region-specific contrasts or omnibus contrast (see Methods: Differential expression analysis), albeit at low magnitudes (mean absolute linear fold difference of autosomal DE genes was 1.158). Consistent with the region-specific nature of brain gene expression and regulation [52–54], regional totals of DEGs were highly variable, from < 10 to thousands of autosomal DEGs. When considering all regions jointly in the omnibus model, 860 DE genes (786 autosomal) were identified, representing 6.1% of analyzed genes (5.8% of autosomal genes, Fig. 1E, F). As expected, the number of DEGs is driven in part by *n*, as the correlation between sample size and number of DEGs is 0.64 (Spearman’s S = 101.68, P-value = 0.02368). The single autosomal DE gene found in all regions was the long, non-coding RNA (lncRNA) *LINC01597*, found to be upregulated in males. This lncRNA is relatively unannotated, but some exons are conserved across closely related species (Supplemental Fig. 2). Surprisingly, we also found a number of chrX genes with male bias, including pseudoautosomal (shared regions of chrX and chrY) genes *PLCXD1*, *ZBED1*, and *ASMTL*, consistent with recent reports for cortex and hippocampus (but not caudate) from an independent dataset [25].

We further demonstrate that there are expression differences between regions, when considering raw counts, SV-adjusted counts, (Fig. 1A) or log_2_FC from differential expression (Fig. 1G, H). Most pronounced are perhaps the difference between the cerebellum and the other brain regions, which is consistent with prior studies (e.g., [55]). Based on the number of sex-differential genes and SNRs, we categorized the cerebellum, cortex, nucleus accumbens, putamen and caudate as sex-differential regions (SDRs) and plotted the top 100 variable log_2_FC DE genes (significant in at least one region) principle components (PC) to illustrate this grouping structure (Fig. 1H). To examine which of these 100 most variable genes was driving the differences we plotted their relative contributions to the PCs (Fig. 1I). One gene of interest is *GCSAML*, which was found to be DE (female upregulated) in the cerebellum, and very lowly expressed in all other brain regions. *GCSAML* is known to be involved in the proliferation of B lymphocytes, interestingly *GCSAML* is also highly expressed in the testes and prostate of males [56].

### Replication and meta-analysis of cortical findings with Commonmind Consortium

To confirm these findings were reproducible, we replicated this study using the control samples from another large (n = 278 males, 159 females) postmortem collection (albeit limited to cortex), the Commonmind Consortium (CMC). We observe significant correlation in sex effects across these independent datasets (Fig. 1J), especially when considering the union of their DEGs (Fig. 1K). Using Fisher's method to combine the P-values from both studies identifies a list of high-confidence cortical sex DEG that may be useful for future analyses (Supplemental Table 4).

### Sex DE genes highlight neuronal and immune signatures in male and female brain

**Fig. 2:**
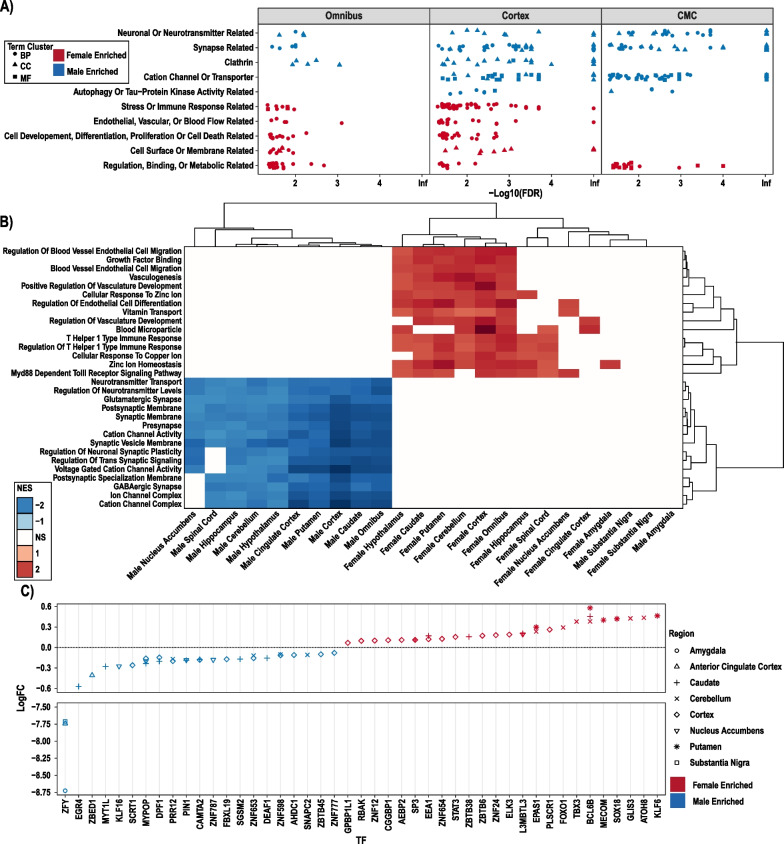
Pathways transcriptionally enriched in the male and female brain. **A** Male, Female, GTEx Cortex, GTEX Omnibus and CMC Cortex upregulated GO terms from FDR significant autosomal genes, shows male-biased and female-biased categories in cortex and omnibus. **B** GSEA plot of all tissues and sexes top 15 mean highest significantly enriched categories’ NES scores from males and females clustered by sample and NES score with white indicating non-significant. GSEA results generally mimic GO results, but there is less replication across female regions, and some regional differences. **C** ChEA3 analysis of DE genes highlights TFs (x-axis) which may be relevant activators that drive DE across regions

After establishing the detectable difference between males and females, we next investigated which biological pathways differ between the sexes. For each region we conducted Gene Ontology (GO) analysis of FDR DE genes per sex supplemental Figs. 3–7. For cortex and omnibus models we followed up this analysis with semantic similarity clustering to reduce redundancy of terms and increase presentability (Fig. 2A, full GO analyses in supplemental Figs. 3–7). Males showed upregulation of neuronal pathways (which reproduced across both datasets) while females showed an upregulation in immune, vascular, and endothelial cell signatures, consistent with a recent report in independent brain data [25].

Furthermore, although many of these ontologies were shared across regional and omnibus models (Fig. 2B), some appeared to be region-specific. For example, male upregulated genes in the nucleus accumbens showed mitochondrial signature and lacked the male neuronal signature found in other regions (Supplemental Fig. 7C). Also, regions with low SNR values or a small number of DE genes did not reflect the general GO enrichments above. Interestingly, the cerebellum did not share the ontology findings of other regions despite having the highest regional SNR and a large number of DE genes. Indeed, essentially no terms were enriched for male upregulated genes, and female DE genes yielded terms with only vague resemblance to the immune and endothelial signatures found in other regions. Overall, these findings provide additional evidence that indicate regional differences in sexually heterogeneous gene expression, with the cerebellum in particular being unique in its expression profile.

Next, we investigated potential drivers of the DEGs in the different regions by examining transcription factors (TFs) whose targets were significantly enriched in our DEG lists with the ChEA3 tool [44]. To identify potential regulators, we subsetted to only transcriptional activators that were themselves DE in each region. We found several high-confidence TFs, which may play a role in modulating transcription in each region (Fig. 2C, Supplemental Table 5). Notably, the female enriched gene, *BCL6B*, is predicted to be involved in inflammatory response, which suggests a mechanism for why many inflammatory genes are upregulated in females across regions.

### Sex-specific signatures are mainly driven by differences in cell type states

Our GO results suggested many of the sex DE genes corresponded to particular cell types. We first sought to confirm this with cell type focused tools and then to interpret these results leveraging recent collections of single-nucleus RNA-sequencing (snRNA-seq) data.

**Fig. 3:**
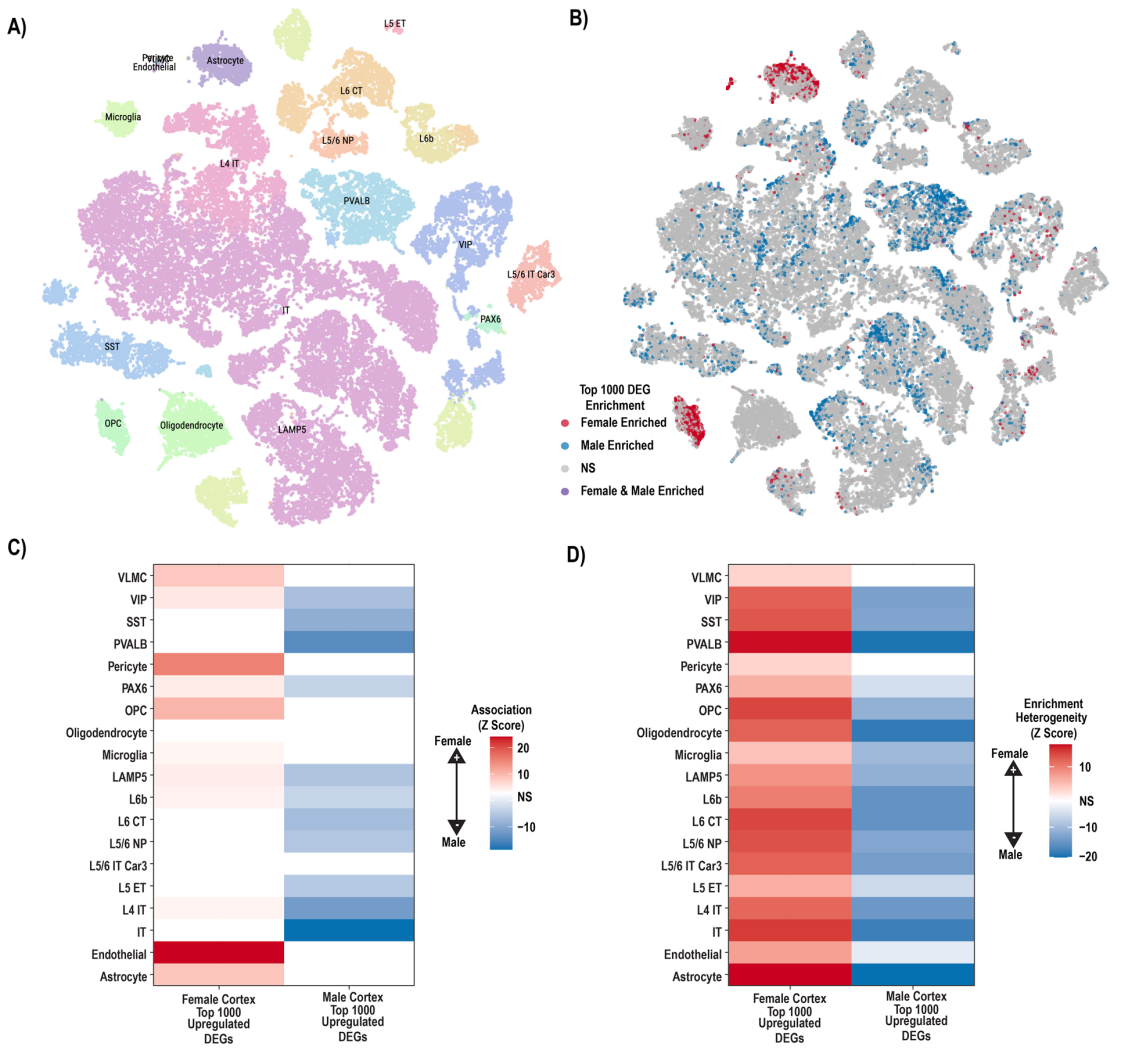
Integration with single nucleus data suggests sex biases in cell states. **A** tSNE illustrating subclasses of single nuclei as identified by Hodge et al. [45]; **B** male upregulated genes (blue) and female upregulated genes (red) map to individual nuclei in distinct clusters. **C** Significance of *overall* enrichment for sex DE genes within each Allen Atlas subclass where white indicates non-significant enrichment after Bonferroni correction. **D** Significance of *heterogeneity* in sex DE gene enrichment for each Allen Atlas subclass where white represents non-significant enrichment after Bonferroni correction

To confirm DE enrichment in the cell types suggested by ontology analysis, we intersected DE genes to mouse brain cell type markers using Cell type-Specific Expression Analysis (CSEA) [57]. While mouse brain cell type markers are not ideal to assess cell identity, conserved basic biological pathways and homologous genes shared between species can allow us an additional estimate of cell identity. CSEA again revealed a neuronal signature in males and a glial/immune signature in females (Supplemental Fig. 9). However, these initial analyses utilized tools based on a limited number of purified cell type RNA rather than true single cell measures [58]. Furthermore, this observation of sex DE gene enrichment in cell type-specific genes/ontology terms could be driven by either sex differences in cellular abundance or by a sex bias in cellular states within each cell type. Thus, to more rigorously identify cell types enriched for cortical sex DE genes across a larger number of cell types, and to clarify whether these enrichments represent abundance or state differences, we leveraged the Allen Brain Atlas snRNA-seq data from the adult human cortex [45] (Fig. 3A) with the single cell disease relevance score (scDRS) tool [59]. The scDRS tool links individual cells to a gene list by generating an empirical P via a permutation test, essentially, this tool estimates which cell types are most involved with a given set of genes. Our aim was to identify whether or not there was an overall enrichment of sex DEGs per cortical cell type, and whether that enrichment was heterogeneous. If sex DE genes were reflective of a particular state (or subpopulation) of a cell type, enrichment heterogeneity would be expected (e.g. the sex enriched genes would be found in cells in just a subpart of a given type), whereas homogeneous enrichment would be expected of a cell type with sex differences in cell type proportion (sex-enriched genes would be found evenly throughout all cells of a type). One example of a distinct transcriptional state/subpopulation is activated microglia, which have a different expression profile than inactive microglia [60].

To test this, individual cells were scored for weighted enrichment in the top 1000 autosomal sex DE genes in cortex for each sex (Fig. 3B), followed by enrichment and heterogeneity testing for each cell type using the single cell enrichment scores [59] (Fig. 3C, D) in the Allen Brain Atlas snRNA-seq data from the adult human cortex [45]. In brief, the scDRS algorithm works to test for enrichment in a cell type by generating a null distribution of randomly selected cells, each ranked with a Z-score according to their relative expression of the input set of genes; an enriched cell type has more cells that are in the upper quantiles of expression Z-scores than would be expected from the generated null distribution. Male cortex upregulated genes were most strongly enriched in several neuronal lineages, but heterogeneously so; top *Z-*scaled male enrichments highlighted intratelencephalic neurons (13% of cells, *Z* > 18, Bonferroni-adjusted heterogeneity *p* < 0.05) and *PVALB*-expressing neurons (39% of cells, Z > 14, Bonferroni-adjusted heterogeneity *p* < 0.05) (Fig. 3B–D). Female upregulated genes from cortex were most strongly enriched in 40% of single astrocytes and 63% of single oligodendrocyte progenitors (Fig. 3B, C; *Z* scaled enrichment scores > 10 and 8, respectively, both Bonferroni-adjusted *P* < 0.05), each also with significant inter-cell heterogeneity (Bonferroni-adjusted *P-value*s < 0.05) (Fig. 3D). These findings strongly suggest that it is particular states of most cortical neuron types, astrocytes, and oligodendrocyte precursors that drive the overall sex-differential gene expression seen in bulk RNA-seq. Complete results are in Supplemental Table 6. Females upregulated genes also showed enrichment in pericytes and endothelial cells, consistent with GO and GSEA analyses. To confirm these results were not spurious, we selected a random set of brain-expressed genes, arbitrarily assigned P-values from the real DEG lists and repeated both analyses. We found no significant overlap with either cell type or heterogeneity measures using this procedure.

### Lack of molecular predisposition, but increased expression of autism risk genes in male brain

We next examined how sex DE patterns relate to neurological disorders with sex biases in diagnosis, with two non-exclusive approaches. First, we examined prior case–control RNA-seq data to determine if male brains exhibit greater similarity to autism cases rather than controls, indicating a male molecular predisposition towards an autism-like state [30, 48], while asking if female brains lean more towards an Alzheimer’s-like state. Secondly, we investigated whether there is any sex bias in the expression of Alzheimer’s- or autism-associated genes in control individuals.

#### Comparison of case–control DEGs to Sex DEGs

**Fig. 4:**
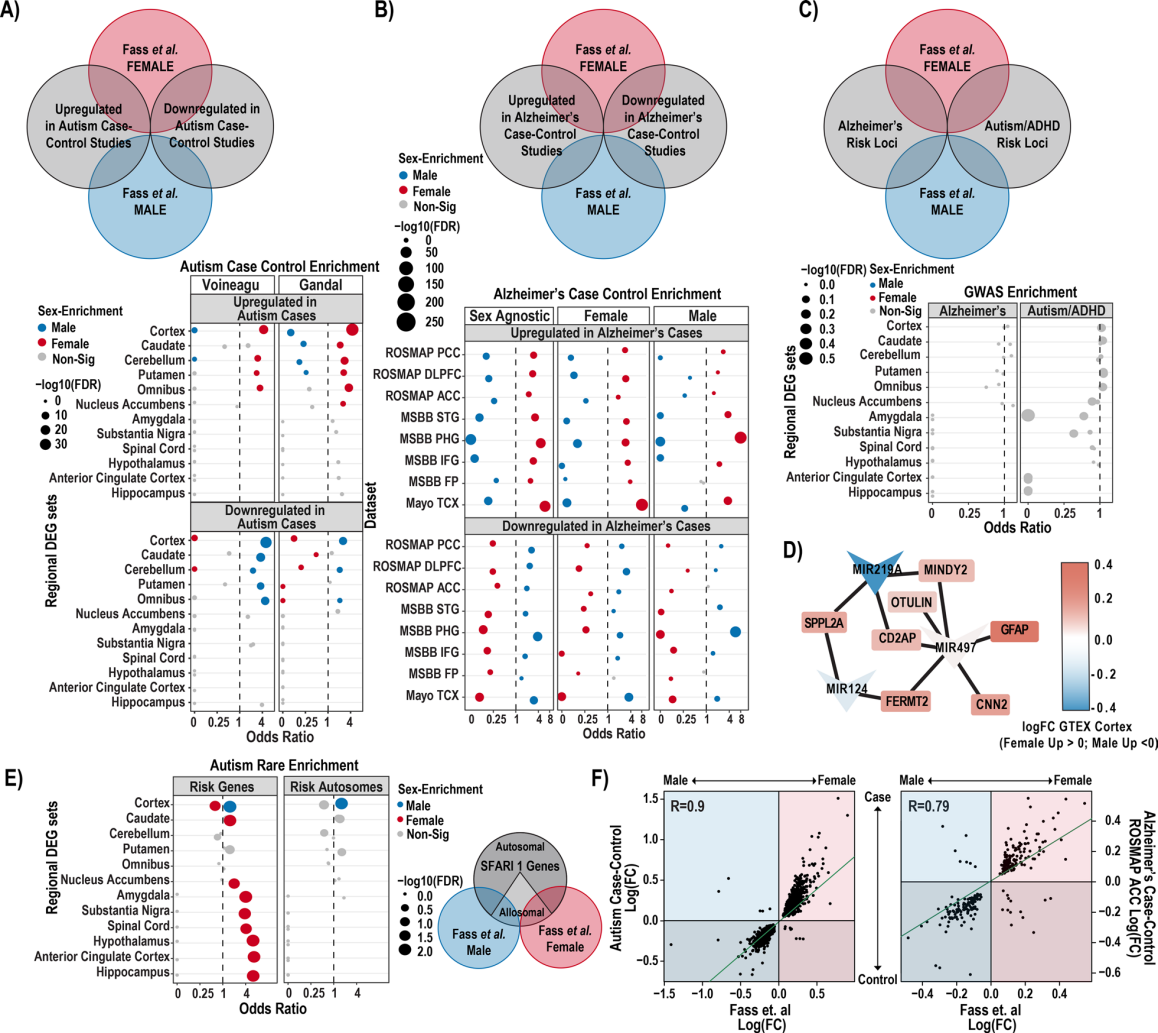
Overlap of sex differences in gene expression to postmortem case–control differences and disease gene sets. **A** Diagram showing comparisons made (top). Autism significantly up- and down-regulated genes sets from case–control studies overlapped with GTEx cortex male and female DEGs (bottom). **B** Diagram outlining general comparisons as in **A** (top). GTEx cortex male and female DEGs overlapped with male, female and sex agnostic Alzheimer's up and downregulated genes sets from cortical samples (bottom). **C** Diagram as in **A**, **B** (top). GWAS Alzheimer’s and autism/ADHD risk loci overlapped with male and female DEGs, shows no significant enrichment (bottom). **D** Male DE microRNAs targets found in GWAS Alzheimer’s risk genes sets. **E** Diagram showing various dataset overlapping performed (right). Autism risk genes from SFARI genescore 1 both including and excluding allosomal genes, shows significant male enrichment in the cortex (left). **F** Scatterplot displaying the relationship between the logFCs of case–control data (y-axes, Autism—left and representative Alzheimer’s—right) and our sex DEG analysis (x-axes). Regression lines are shown in green and Pearson’s R values are displayed in the top right corner of each graph. If no genes overlap, then the odds ratio is 0

Female enriched genes were generally higher in autism cases than controls, contrary to a molecular predisposition hypothesis. This was true across two gene sets from autism case–control studies (Fig. 4A). Likewise, we found that autism downregulated cortex genes relative to controls were found to be enriched in the male upregulated cortex DEG set.

In addition to looking at gene overlap in previous bulk RNA-seq autism case–control studies, we also examined the gene overlap in Alzheimer's bulk RNA-seq case–control studies. We chose an analysis [50] that used multiple cohorts of Alzheimer’s donors from different institutions and analyzed the data using several statistical models. We chose the cortical samples for each of these cohorts and used three of the analyses: diagnosis only, diagnosis by male sex only, and diagnosis by female sex only. Regardless of which cortical region examined, or which analysis used, we saw the same, clear pattern emerge. Genes upregulated in Alzheimer’s donors significantly overlap with genes upregulated in female cortex, and genes upregulated in control donors significantly overlap with male cortex (Fig. 4B). This provides evidence that females have a molecular predisposition for an Alzheimer’s-like state.

In addition to overlapping the gene significant lists in a categorical manner, we also tested the correlation between our neurotypical sex differences and case vs. control status using a quantitative approach. Indeed, correlating the log fold-changes in sex differential genes with the log-fold changes of case vs. control studies of autism and Alzheimer’s were consistent with the categorical analyses. Specifically female enriched transcripts defined in neurotypical brains were also enriched in both Alzheimer’s cases and autism cases relative to their controls (Fig. 4F). The correlations of log fold-changes in these analyses were quite high (> 0.79) across all case–control comparisons, and were similarly high whether or not sex chromosomes were included (not shown). Furthermore Y-chromosome genes were not found as case–control differential genes, suggesting there was not a cryptic unaccounted for sex bias in those previously published studies that could explain this effect.

#### Common risk variants

One possible mechanism by which sex differences arise in diseases with complex (i.e. polygenic, common variant mediated) heritability is through variant-mediated perturbations to baseline sex-differential gene expression patterns. For example, a set of genes expressed in a given tissue and linked to nearby common risk variants through genome-wide association studies (GWAS) could be upregulated or downregulated as consequences of risk alleles, altering cell fate or state when collective effects surpass some threshold. Sex differences in gene expression could either buffer against or predispose toward reaching a collective effect threshold in a given pathway or cell type. For example, if disease *D* risk loci cumulatively downregulates pathway *Z*, and pathway *Z* is upregulated in healthy females in the disease tissue, then females should be resilient to genetic risk factors acting through pathway *Z*. Indeed, such mechanisms have been speculated to account for differences in autoimmune disease rates between men and women [61, 62]. Therefore, by looking at which common polymorphism loci show some type of nominally significant contribution to risk, we can see if any up or downregulated genes overlap and possibly identify mechanisms for risk.

We first tested whether any of the analyzed regions’ sex differential genes were enriched for genes associated with Alzheimer’s or a joint analysis of ADHD/autism by prior and recent GWAS studies [11, 12]. No significant enrichment of sex DE genes were found in autism or Alzheimer’s GWAS loci genes, however, GWAS loci each usually contain several genes, only a subset of which are related to the condition (Fig. 4C). Thus, we conducted a preliminary analysis of the subset of GWAS loci genes that did overlap with sex DEGs, to see if any patterns emerged. One interesting overlap was with a lncRNA found near the critical Alzheimer’s gene *APP–AP00023.1*—which was significantly upregulated in female GTEX omnibus and cortex. 15 additional Alzheimer's risk genes significantly upregulated in female cortex were *RPS27L*, *SPPL2A*, *PRKD3*, *MINDY2*, *FERMT2*, *TMEM106B*, *FAM96A*, *RAB8B*, *ABCA1*, *CD2AP*, *OTULIN*, *FOXF1*, *CNN2*, *GFAP*, and *GDPD3*. To further investigate GWAS loci that did overlap with our DEGs we conducted a downstream analysis and identified enrichment for targets of the miRNA, *MIR219A2: ABCA1*, *MINDY2*, *SPPL2A*, and *CD2AP*. *MIR219A2* was upregulated in male cortex and omnibus at nominal significance (FDR 0.063), while the aforementioned targets were upregulated in females at corrected significance. This relationship is consistent with the repressive role of miRNAs, and suggests that *MIR219A* may confer a protective effect against Alzheimer’s by repressing these targets (Fig. 4D). One study has provided evidence that MIR219A overexpression helps regulate the differentiation of oligodendrocyte precursor cells (OPCs) into oligodendrocytes, promotes remyelination, and improves cognitive function [63]. Additionally, autism GWAS loci genes intersecting male upregulated genes from GTEX cortex were enriched for putative target genes of the transcription factor and androgen receptor (AR) coregulator [64] *ZBTB7A*. *ZBTB7A* fell just short of corrected significance for upregulation in male cortex (FDR = 0.06), but could hint at a similar mechanism for why this set of genes is higher in male brain.

#### Rare variant-implicated autism genes

We also tested for enrichment of rare variant-implicated autism genes in the regional sex DEGs lists. We utilized the SFARIGene database [31], specifically genes with high-confidence of playing a role in autism, denoted as genescore 1 (nearly all of which cause neurodevelopmental syndromes with high penetrance of both autism and Intellectual Disability). We found there to be a significant overlap of these high confidence autism risk genes across many of the regional sex DEG sets. Across most brain regions the autism risk genes were overlapped with genes expressed higher in females (except cortex, which showed a male bias). However, this likely reflects the fact that many of the risk loci are found on the X chromosome (21 of the 211 autism genes were on the X chromosome), which are more often upregulated in females. To remedy this bias, we repeated this analysis excluding all allosomal genes (Fig. 4E). We observed the male cortex was still enriched for autism risk genes regardless of allosomal exclusion (Fig. 4E). It should also be noted that when using the SFARIGene database list as it existed at the time of the Werling 2016 paper, we replicate the findings of Werling 2016 and find no sex DEG sets to be significantly enriched. Finally, for Alzheimer’s, relatively few rare loss of function variants have been robustly associated with the disease, precluding a similar analysis.

To ensure that our significant overlap of male DEGs with rare variant-implicated autism genes was not simply reflecting the higher male expression of neuronal genes, combined with the known bias of autism genes towards neuronal expression. To test this, we generated 1000 random gene sets of the same number and expression level in snRNA-seq neuronal data, as the autism gene list and ran enrichment testing in the male cortex DEGs. We did find these random neuron-biased lists were slightly enriched in male brain DEGs (mean odds ratio of ~ 1.2), yet they rarely matched the odds ratio observed in the true autism gene list (Supplemental Fig. 10).

## Discussion

This study comprehensively examines adult sex differences across brain regions and across the brain as a whole under a single unified model, providing a valuable resource for future reanalyses. Interestingly, with the power of all samples available, more than 5% of genes included in this analysis showed significantly sex-biased expression, albeit often of very low magnitudes in the omnibus model. Interpretation of these results are complex and can vary greatly depending on selected log_2_FC thresholds. Thus, care must be taken in selecting significance and log_2_FC thresholds most relevant to a given line of inquiry or quantitative approaches leveraging all available log_2_FC values should be taken. In addition to the GTEx omnibus model we also present the individual analyses for each brain region (Supplemental Table 1).

Looking across regions, *LINC01597* is a newly identified sex DE gene of particular interest, as it shows similar male-increased expression patterns across all models to that of an allosomal gene despite not having homology to any known Y region (Fig. 1F). This extreme sex bias can be seen in other large human genetic studies [65, 66]. This could be a novel example of a uniquely regulated pseudoallosomal gene that may have important function, considering its high relative expression in the brain [56] and pituitary [33]. The *LINC01597* gene is found near the centromere of chromosome 20 and shows some conservation (Supplemental Fig. 2). Search of *LINC01597* sequence to telomere to telomere (T2T) genome using UCSC BLAT search confirmed no homology to Y genes, ruling out mismapping of Y-derived reads as driving this finding. Considering that some lncRNAs serve as important mediators of sex-specific gene expression, such as *XIST*, and have been associated with increased risk of depression and cancer [67–69], *LINC01597* may merit additional investigation.

From pathway/ontologies analyses, our findings indicate a significant upregulation of genes associated with the Homotypic Fusion and Protein Sorting (HOPS) complex within the male cortex as well as tau protein kinase activity (Fig. 2A). The HOPS complex is recognized for its involvement in autophagosomal activity, a process crucial for removing debris from the cell. Dysregulation of the HOPS complex can potentially lead to neuronal cell death. Notably, if females exhibit a relatively lower HOPS complex expression or a reduction in the amount of tau-kinase activity, these features may provide partial explanations for the higher prevalence of Alzheimer's disease in the female population.

Examination of this data with regards to cell types suggests states of particular cell types may also contribute. We observe evidence of sex-differential cell states in neurons, with males having an upregulation for certain neurotransmitters and synapse formation genes while females show evidence for possessing distinctive vascular, endothelial, and immune signatures. Specifically, our findings contribute evidence to a growing body of knowledge that indicates the female brain has increased levels of immune activity, either via an increased abundance of immune cells (particularly microglia) or via a more active state of these cells [70–73]. Our subsequent analyses incorporating snRNA-seq data suggest that females do not have an increased abundance of microglia cells; rather that these cells are in a different state (Fig. 3C, D). Distinct microglia states are strongly implicated in Alzheimer’s risk and progression through both human genetics and pathology [74, 75]. For example, high-throughput analysis of microglial morphometrics in mice also indicated female microglia are in a more disease-like state, and more rapidly shift into this state in progress of disease models as well [76]. If microglia in the human female brain are also already slightly shifted toward such states, this could enhance the risk of developing disease. This shift would have implications for diseases and disorders beyond Alzheimer’s disease, including Multiple Sclerosis [77].

For the vascular cells (endothelial and pericytes) the analysis suggested there may be more such cells in female brain (Figs. 2A, B, 3C), but the number of these cells in the single cell data is relatively low (< 100) precluding a thorough analysis of heterogeneity of states of these cells. Nonetheless, it would be interesting to assess whether there is more vascularity in the female brain.

There may be an opportunity to consider treatments or risk mitigation approaches that are informed by the sex of the person, although we are unaware of exactly how these sex differences in gene expression are being driven. One possibility is miRNA 219A2, which we observed as being possibly sex-biased. If so, this could be male-protective via degradation of Alzheimer’s implicated transcripts, providing one possible explanation of increased female risk for Alzheimer’s disease. However, other possible mechanisms may include specific TFs, like *BCL6B, SCRT1* highlighted here, or others in the cacophony of regulators that are downstream of the allosomes, via sex hormones, or a nebulous combination of environmental and sociological factors, or a nuanced and complex mixture of all of the above. Thus, the female gene expression bias towards an Alzheimer’s-like state might suggest any molecular predisposition is acting through multiple convergent molecular pathways. It is interesting that Alzheimer’s GWAS signal was not enriched in any particular brain region (Fig. 4), suggesting that the well-documented differences in vulnerability (e.g. the earlier formation of pathology in hippocampus relative to cerebellum), are perhaps not driven by some kind of sex expression by gene interaction in these regions.

Notably, elderly individuals may exhibit an augmented immune response in brain tissue, and since females tend to live longer, it is plausible that the observed immune signature may be caused by data skewed toward older donors. However, to rule out this possibility, we reran the analysis with age as a covariate and the findings remained with regard to the immune signature above. The minor differences to note between the models were that more sex effects were detected with age in the model in particular brain regions, notably limbic regions like the amygdala, accumbens, nigra, and spinal cord. Thus, at least within the age ranges present here, the altered immune response seemed to be a *bona fide* sex difference rather than age effect, though age may interact more with sex in some specific regions.

Somewhat more difficult to interpret is the evidence for neuron transcripts seemingly to be generally upregulated in males. Our snRNA-seq data analysis seems to suggest this sex bias is due to a change in state (Fig. 3). One hypothesis could be that neurons in males brains are regulated by a slightly different proteasomal and hormonal environment, which causes these neuronal pathways to appear differentially expressed, perhaps including the HOPS pathway discussed above.

Unlike our Alzheimer's analysis, our case–control gene expression findings did not support a male molecular predisposition as driving the sex difference in autism prevalence, in contrast to prior work [16]. While the sex DE here was well powered, our data sets sampled an older population, thus differences in either power or age of samples may explain the difference in findings from the prior study. Moreover, the female component of our dataset is largely postmenopausal in age, and would miss gene expression effects of many circulating hormones. Future well-powered studies should test whether this same sex DE pattern holds in younger brains, especially those from ages when autism is diagnosed.

Our male-biased neuronal signature is also in direct contrast to a recent study aggregating sex differences across 46 different RNA-Seq and microarray datasets, which suggested a female-biased neuronal signature [78]. It is hard to directly compare our results as our approaches were different in many parameters. Just a few examples of differences in approach that could explain the differences in findings include (1) whether multiple samples from the same donor were appropriately accounted for statistically in the gene expression model (2) whether brain disease carriers were excluded, (3) whether different regions were analyzed in a single model or separately, (4) whether findings were aggregated across studies, and if the aggregation algorithm accounted for differences in sample size across studies. Perhaps, most interestingly [78], contained numerous younger samples in some collections and even embryonic samples. This would be consistent with the pattern of sex bias changing over age as being the best explanation for the differences. Indeed, one early microarray study covering 20–60 year olds in a more balanced way revealed increasing relative immune signature in females with age [79].

It is interesting that outside of the X chromosome, rare variant causal genes for autism overlap with male-biased sex-differential genes in the cortex. We could interpret the overlap as a greater dependence on each of these genes by the male cortex, though of course any individual carrying these mutations is only losing one of these genes, not the whole set. Postmortem studies of individuals with these rare mutations may be informative in better understanding this result.

### Perspectives and significance

Overall this work provides a robust analysis of adult human RNA expression across multiple brain regions as a resource for future use. These findings highlight differentially expressed genes across several brain regions, with patterns in male- and female-biased genes. Although we chose to investigate primarily Alzheimer’s and autism, this study identifies individual genes and specific pathways to consider when trying to better understand sex biases in many diseases. This work can serve as a resource for further analysis of sex variation in the human brain. We can speculate that Alzheimer's pathogenic immune and endothelial signatures may be driven by baseline sex biases, and future studies should investigate this phenomenon.

## Conclusions

Numerous but small autosomal sex differences in expression exist in all brain regions tested, but especially in the cortex, caudate, putamen, cerebellum and nucleus accumbens. Autosomal genes with enriched expression in males are enriched in neuronal, autophagy, and tau-protein kinase pathways. Autosomal genes with enriched expression in females are enriched with immune system, endothelial, and vascular pathways. Integration with snRNA-seq data sets suggest these differences are more likely related to cell state differences than cell number differences. The female cortex shows an enrichment of genes expressed in Alzheimer’s disease brains. The male brain does not follow an autism molecular predisposition hypothesis, rather results align better with an autism female protective effect hypothesis. This work can be used as a resource for specific genes to consider when trying to better understand sex biases in health and disease.

### Supplementary Information

Supplementary Material 1. Supplemental Figure 1: Mean variance trend. GTEx genes that passed filtering steps and their mean variance trend; shows most genes have a squared standard deviation of .5 to 1.5. Supplemental Figure 2: Conservation of LINC01597. UCSC genome browser track of *LINC01597.* Phylop and alignment tracks show conservation of exons. Segmental duplication track shows there are duplications of some of this region on other autosomes, but no duplication mapping to the Y chromosome. Adapted from UCSC browser [80]. Supplemental Figure 3: Gene Ontology plot of Omnibus results. **A**) Male Omnibus DEG autosomal genes significant (FDR < .05) GO enrichment term clusters and FDR value. **B)** Female Omnibus DEG autosomal genes significant (FDR < .05) GO enrichment term clusters and FDR value. Supplemental Figure 4: Gene Ontology plot of Cortex results. **A**) Male Cortex DEG autosomal genes significant (FDR < .05) GO enrichment term clusters and FDR value. **B)** Female Cortex DEG autosomal genes significant (FDR < .05) GO enrichment term clusters and FDR value. Supplemental Figure 5: Gene Ontology plot of Putamen results. **A) **Male Putamen DEG autosomal genes significant (FDR < .05) GO enrichment term clusters and FDR value. **B)** Female Putamen DEG autosomal genes significant (FDR < .05) GO enrichment term clusters and FDR value. Supplemental Figure 6: Gene Ontology plot of Caudate results. **A) **Male Caudate DEG autosomal genes significant (FDR < .05) GO enrichment term clusters and FDR value. **B)** Female Caudate DEG autosomal genes significant (FDR < .05) GO enrichment term clusters and FDR value. Supplemental Figure 7: Gene Ontology plot of Cerebellum and Nucleus Accumbens results. **A) **Male Cerebellum DEG autosomal genes significant (FDR < .05) GO enrichment term clusters and FDR value. **B)** Female Cerebellum DEG autosomal genes significant (FDR < .05) GO enrichment term clusters and FDR value **C) **Male Nucleus Accumbens DEG autosomal genes significant (FDR < .05) GO enrichment term clusters and FDR value. Supplemental Figure 8: Cell type-Specific Expression Analysis suggests enriched glial signature in female cortex and neuronal signature in male cortex. **A) **Female omnibus protein coding genes using CSEA tool at a FDR .05 threshold, shows weak enrichment of OPCs. **B)** Male omnibus protein coding genes using CSEA tool at a FDR .05 threshold, shows enrichment for several classes of neurons. **C)** Female cortex protein coding genes using CSEA tool at a FDR .025 threshold, shows enrichment for several classes of brain immune cell types, as well as strong enrichment for OPCs. **D)** Male cortex protein coding genes using CSEA tool at a FDR .025 threshold, shows enrichment for layer 5b and 5a neuron subtypes. Figures generated by CSEA too [57]. Supplemental Figure 9: Odds ratio distribution of SFARI CPM matched neuron expressed genes. OR distribution of enrichment of random genesets with a similar CPM distribution to SFARI genescore 1 genes (vertical line) in postmortem cortex neuron data from the Allen Brain Atlas [45]. Shows that enrichment of rare variant genes in male cortex is not due to the neuronal biased male signature alone.

Supplementary Material 2. Supplemental Table 1. Table of complete results of GTEx DE analysis. For each contrast included in the analysis (both region and omnibus) each genes logFC, P-value, and gene related metadata.

Supplementary Material 3. Supplemental Table 2. Table of complete results of CMC DE analysis. For each gene included in the analysis, logFC, P-value, average expression and gene related metadata.

Supplementary Material 4. Supplemental Table 3. Table of complete results of GSEA analysis of GTEx data. For each sex, for each region, all the enriched gene set categories along with their NES scores, P-values, gene set size and other key information.

Supplementary Material 5. Supplemental Table 4. Complete results of meta analysis, combining P-values from GTEx DE analysis and CMC DE analysis for Cortex. For each gene included in both CMC and GTEx analyses that agree on direction of effect, combined P-value, and gene associated meta are available.

Supplementary Material 6. Supplemental Table 5. Results from ChEA3 analysis. For each regional DEG set a list of the most enriched activators, along with their mean rank, and gene information pulled from the DE analysis.

Supplementary Material 7. Supplemental Table 6. Complete results from scDRS analysis. Complete scDRS enrichment results for Allen brain atlas cortex single-nucleus data, including male and female enrichments for cell sub-class and cortical layers.

Supplementary Material 8. Supplemental Table 7. List of samples used in GTEX analysis. Includes GTEX sample and donor ID, sex and age bracket.

Supplementary Material 9. Supplemental Table 8. Design matrix with SVA adjustment variables. The design matrix used in limma analysis with all values used for surrogate variable analysis adjustments.

## Data Availability

Availability of raw data can differ depending on its status of public or authorized use only. Authorized material is available on gtexportal.org, and synapse.org. Public data is available on gtexportal.org, gene.sfari.org, and several published works as cited throughout the paper. Code used in running this analysis is available at https://bitbucket.org/jdlabteam/gtex_peper_final_code/src/main/. All analyzed results are included in this publication.
