# Supplementary material for: Relationship between sex biases in gene expression and sex biases in autism and Alzheimer’s disease
Source: Biol Sex Differ. 2024 Jun 7;15:47. doi: 10.1186/s13293-024-00622-2 (PMC11157820; doi:10.1186/s13293-024-00622-2)
Supplement: Supplementary file 1 — Supplementary Material 1. Supplemental Figure 1: Mean variance trend. GTEx genes that passed filtering steps and their mean variance trend; shows most genes have a squared standard deviation of .5 to 1.5. Supplemental Figure 2: Conservation of LINC01597. UCSC genome browser track of LINC01597. Phylop and alignment tracks show conservation of exons. Segmental duplication track shows there are duplications of some of this region on other autosomes, but no duplication mapping to the Y chromosome. Adapted from UCSC browser [80]. Supplemental Figure 3: Gene Ontology plot of Omnibus results. A) Male Omnibus DEG autosomal genes significant (FDR < .05) GO enrichment term clusters and FDR value. B) Female Omnibus DEG autosomal genes significant (FDR < .05) GO enrichment term clusters and FDR value. Supplemental Figure 4: Gene Ontology plot of Cortex results. A) Male Cortex DEG autosomal genes significant (FDR < .05) GO enrichment term clusters and FDR value. B) Female Cortex DEG autosomal genes significant (FDR < .05) GO enrichment term clusters and FDR value. Supplemental Figure 5: Gene Ontology plot of Putamen results. A) Male Putamen DEG autosomal genes significant (FDR < .05) GO enrichment term clusters and FDR value. B) Female Putamen DEG autosomal genes significant (FDR < .05) GO enrichment term clusters and FDR value. Supplemental Figure 6: Gene Ontology plot of Caudate results. A) Male Caudate DEG autosomal genes significant (FDR < .05) GO enrichment term clusters and FDR value. B) Female Caudate DEG autosomal genes significant (FDR < .05) GO enrichment term clusters and FDR value. Supplemental Figure 7: Gene Ontology plot of Cerebellum and Nucleus Accumbens results. A) Male Cerebellum DEG autosomal genes significant (FDR < .05) GO enrichment term clusters and FDR value. B) Female Cerebellum DEG autosomal genes significant (FDR < .05) GO enrichment term clusters and FDR value C) Male Nucleus Accumbens DEG autosomal genes significant (FDR < .05) GO enrichment term clu [file 13293_2024_622_MOESM1_ESM.docx]

| **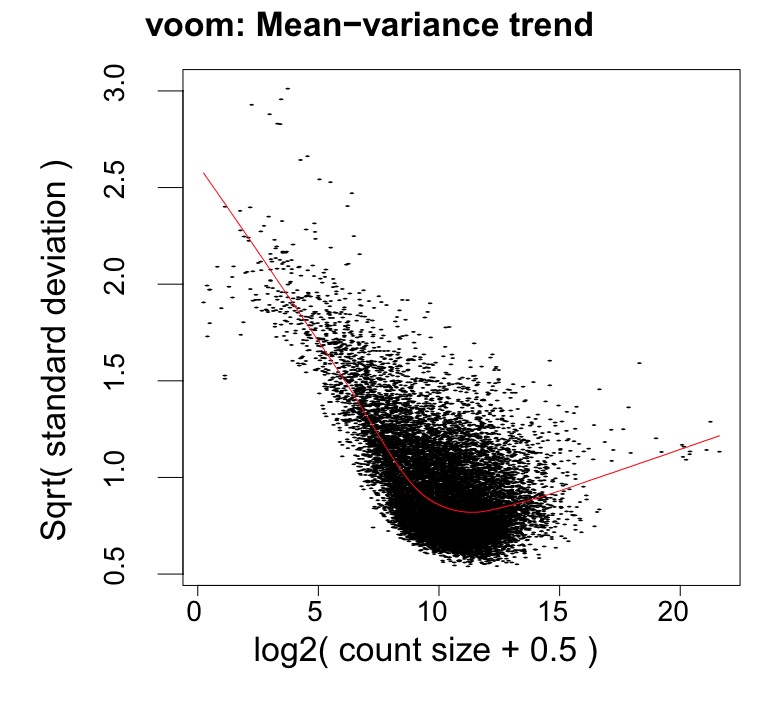** |
| --- |
| **Supplemental Figure 1 Mean variance trend**  GTEx genes that passed filtering steps and their mean variance trend; shows most genes have a squared standard deviation of .5 to 1.5. |

| 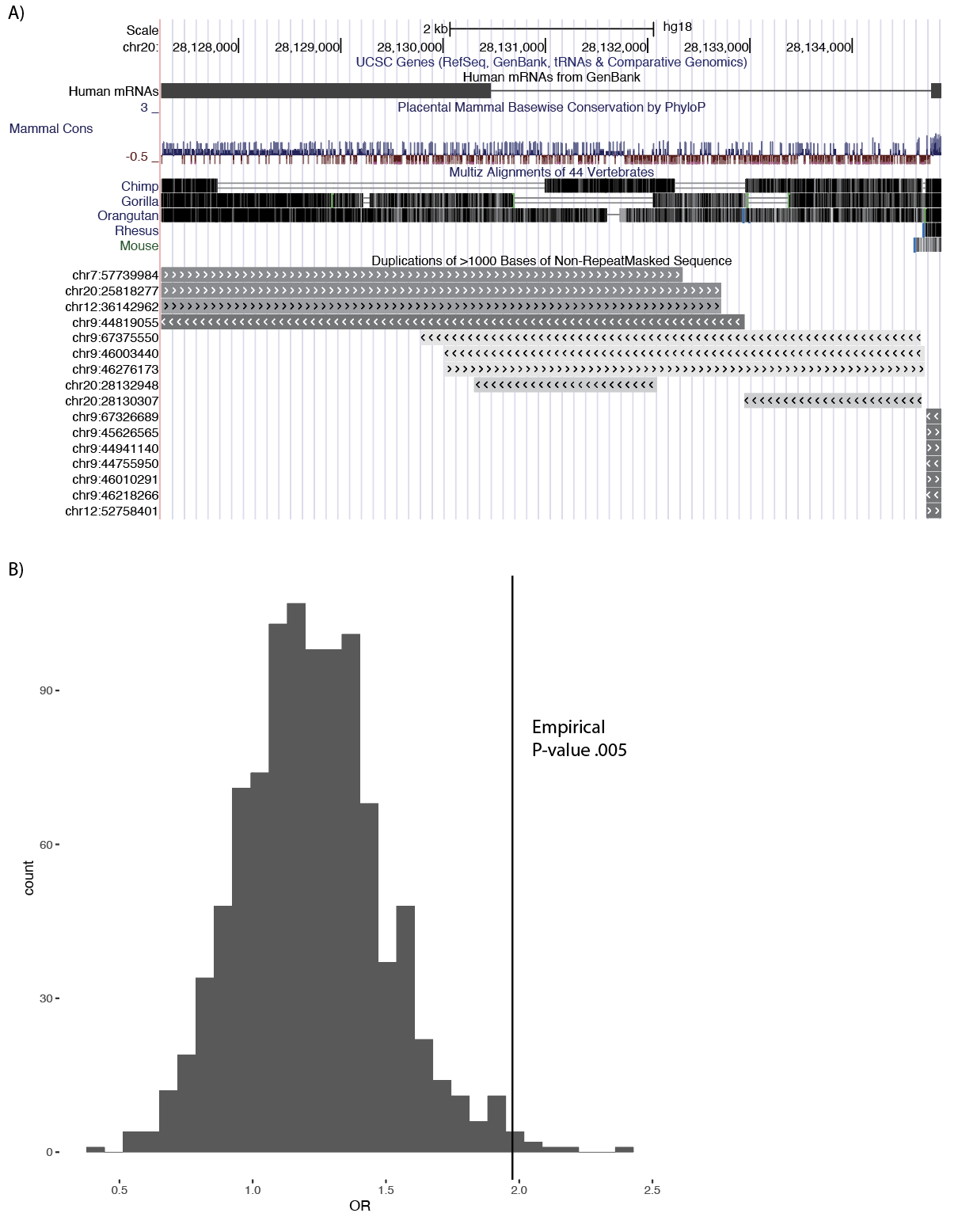 |
| --- |
| **Supplemental Figure 2: Conservation of LINC01597**  UCSC genome browser track of *LINC01597.* Phylop and alignment tracks show conservation of exons. Segmental duplication track shows there are duplications of some of this region on other autosomes, but no duplication mapping to the Y chromosome. Adapted from UCSC browser [(80)](https://www.zotero.org/google-docs/?gsOito). |

| 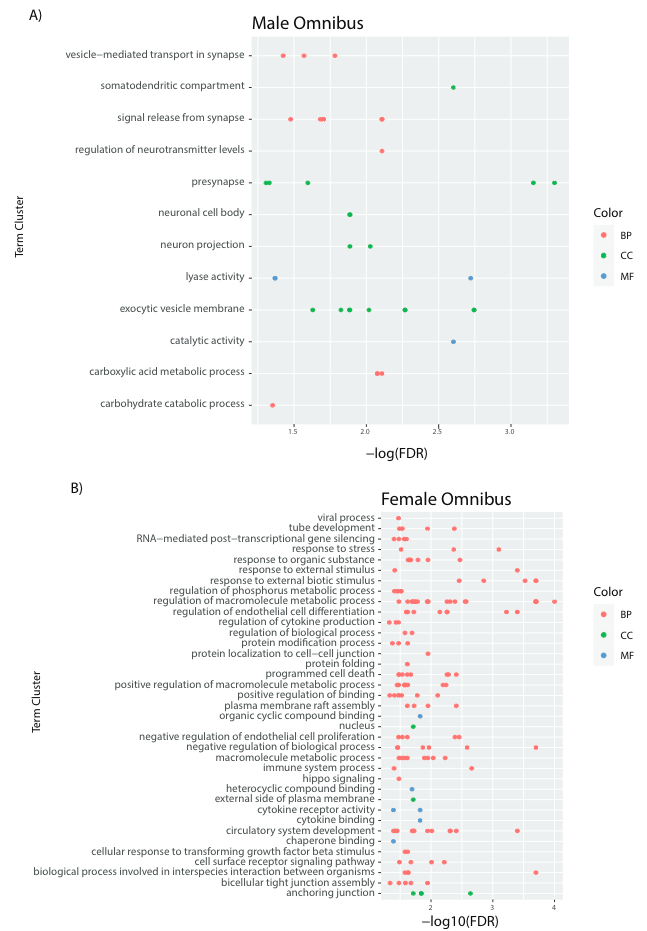 |
| --- |
| **Supplemental Figure 3: Gene Ontology plot of Omnibus results**  **A)** Male Omnibus DEG autosomal genes significant (FDR < .05) GO enrichment term clusters and FDR value **B)** Female Omnibus DEG autosomal genes significant (FDR < .05) GO enrichment term clusters and FDR value |

| 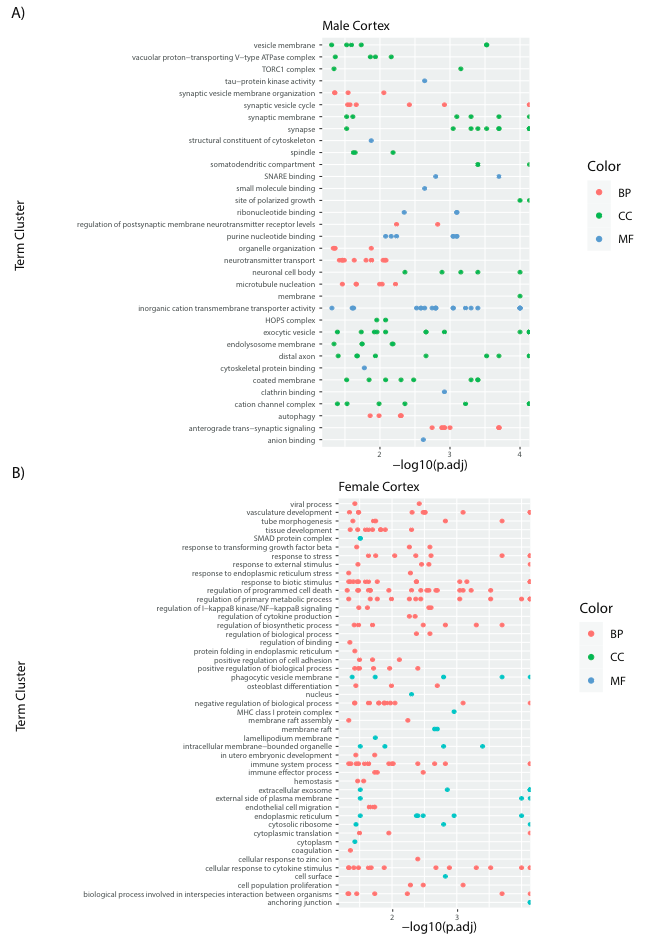 |
| --- |
| **Supplemental Figure 4: Gene Ontology plot of Cortex results**  **A)** Male Cortex DEG autosomal genes significant (FDR < .05) GO enrichment term clusters and FDR value **B)** Female Cortex DEG autosomal genes significant (FDR < .05) GO enrichment term clusters and FDR value |

| 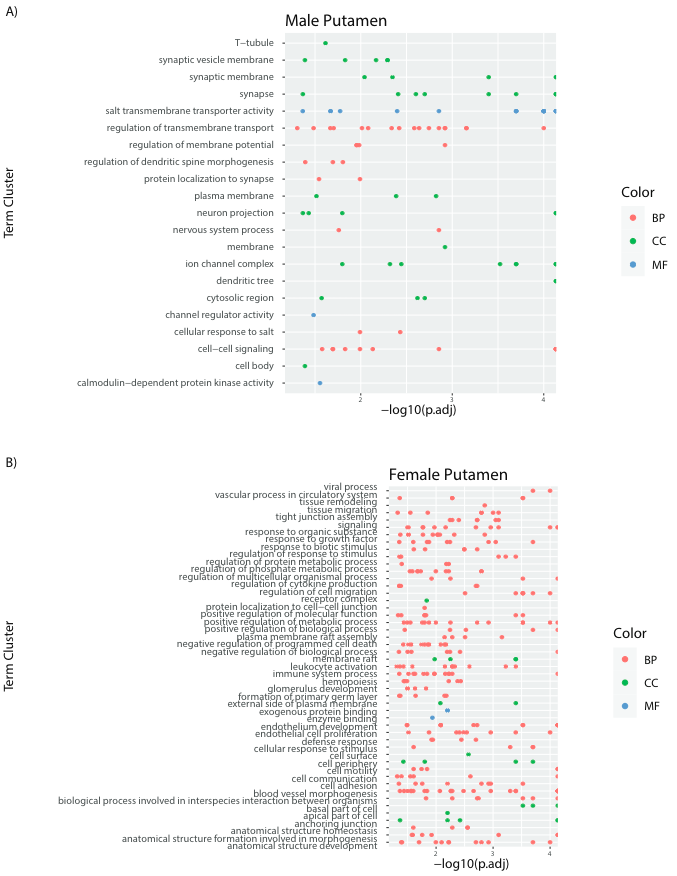 |
| --- |
| **Supplemental Figure 5: Gene Ontology plot of Putamen results**  **A)** Male Putamen DEG autosomal genes significant (FDR < .05) GO enrichment term clusters and FDR value **B)** Female Putamen DEG autosomal genes significant (FDR < .05) GO enrichment term clusters and FDR value |

| 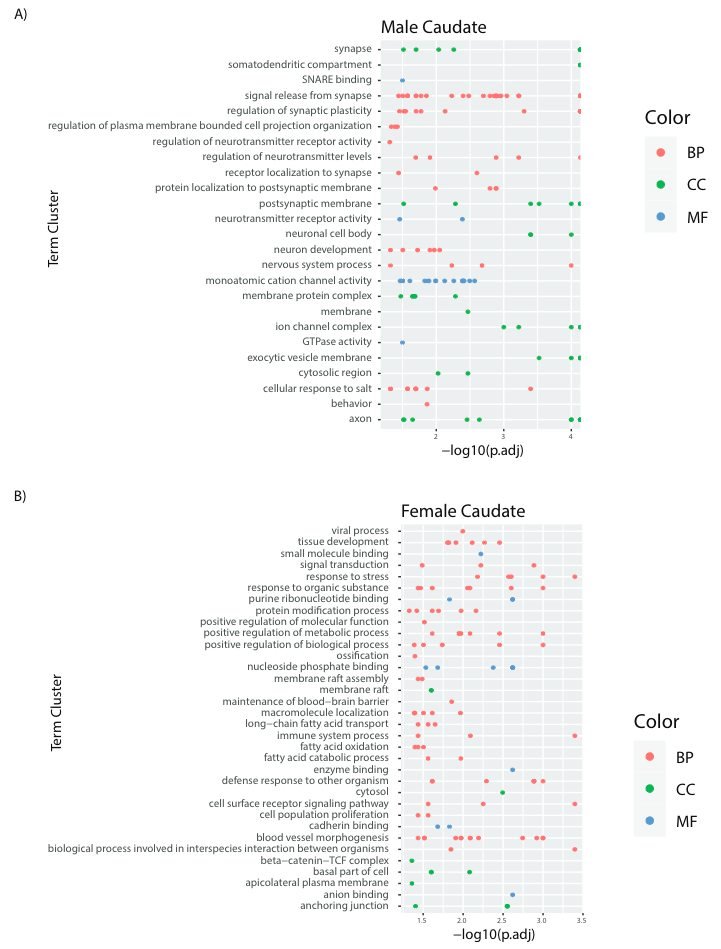 |
| --- |
| **Supplemental Figure 6: Gene Ontology plot of Caudate results**  **A)** Male Caudate DEG autosomal genes significant (FDR < .05) GO enrichment term clusters and FDR value **B)** Female Caudate DEG autosomal genes significant (FDR < .05) GO enrichment term clusters and FDR value |

| 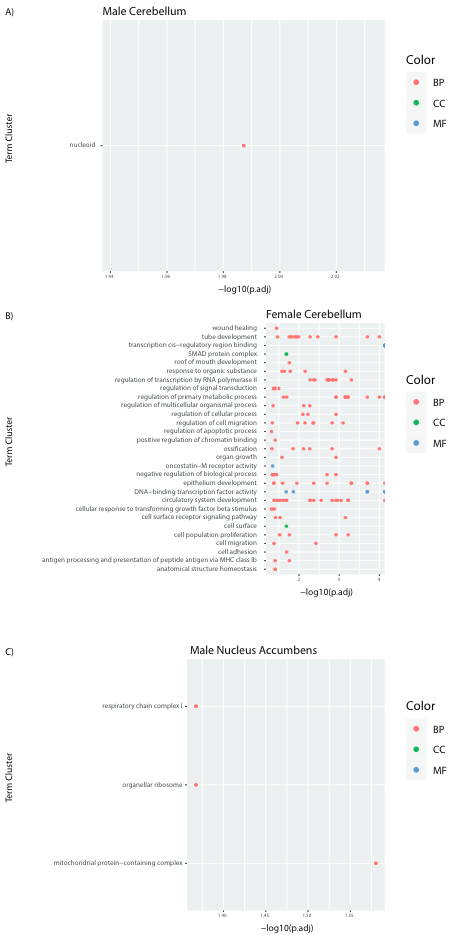 |
| --- |
| **Supplemental Figure 7: Gene Ontology plot of Cerebellum and Nucleus Accumbens results**  **A)** Male Cerebellum DEG autosomal genes significant (FDR < .05) GO enrichment term clusters and FDR value **B)** Female Cerebellum DEG autosomal genes significant (FDR < .05) GO enrichment term clusters and FDR value **C)** Male Nucleus Accumbens DEG autosomal genes significant (FDR < .05) GO enrichment term clusters and FDR value |

| 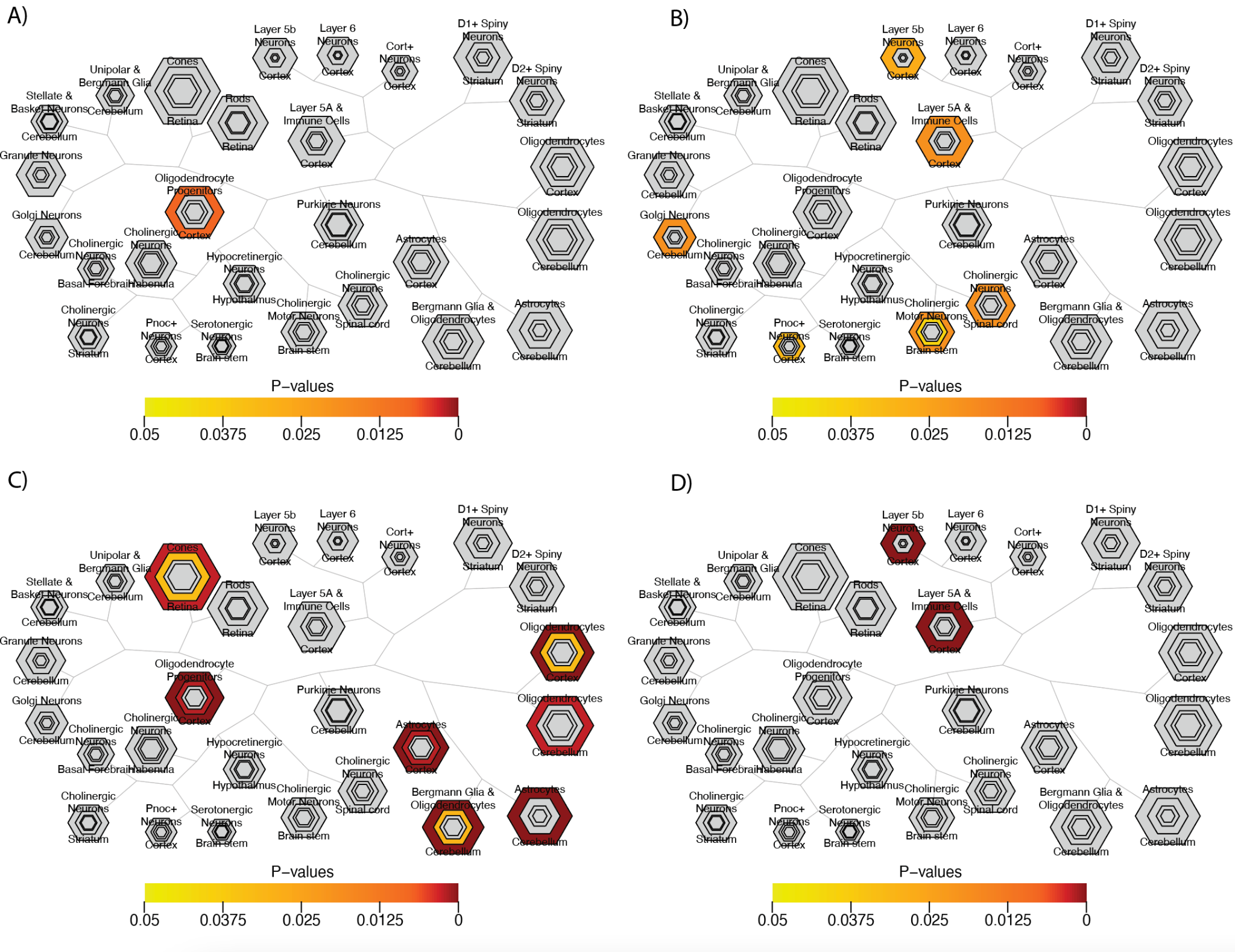 |
| --- |
| **Supplemental Figure 8: Cell type-Specific Expression Analysis suggests enriched glial signature in female cortex and neuronal signature in male cortex. A)** Female omnibus protein coding genes using CSEA tool at a FDR .05 threshold, shows weak enrichment of OPCs. **B)** Male omnibus protein coding genes using CSEA tool at a FDR .05 threshold, shows enrichment for several classes of neurons. **C)** Female cortex protein coding genes using CSEA tool at a FDR .025 threshold, shows enrichment for several classes of brain immune cell types, as well as strong enrichment for OPCs. **D)** Male cortex protein coding genes using CSEA tool at a FDR .025 threshold, shows enrichment for layer 5b and 5a neuron subtypes. Figures generated by CSEA too[(57)](https://www.zotero.org/google-docs/?M77WIl). |

| 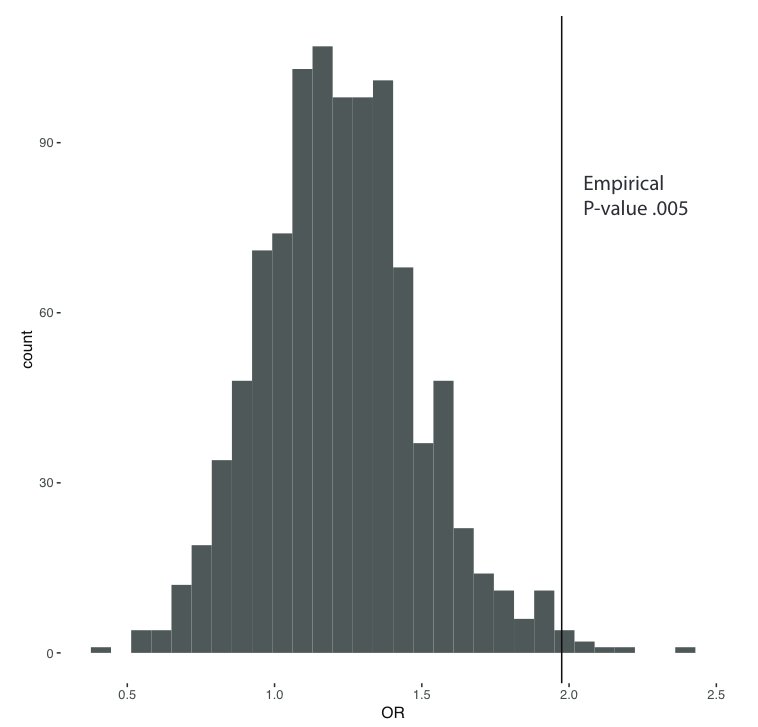 |
| --- |
| **Supplemental Figure 9: Odds ratio distribution of SFARI CPM matched neuron expressed genes**  OR distribution of enrichment of random genesets with a similar CPM distribution to SFARI genescore 1 genes (vertical line) in postmortem cortex neuron data from the Allen Brain Atlas [(45)](https://www.zotero.org/google-docs/?Acoh0E). Shows that enrichment of rare variant genes in male cortex is not due to the neuronal biased male signature alone. |
